# Brain Metastases From Differentiated Thyroid Carcinoma: A Retrospective Study of 22 Patients

**DOI:** 10.3389/fendo.2021.730025

**Published:** 2021-09-16

**Authors:** Tong Wu, Zan Jiao, Yixuan Li, Jin Peng, Fan Yao, Weichao Chen, Ankui Yang

**Affiliations:** Department of Head and Neck Surgery, Sun Yat-sen University Cancer Center, State Key Laboratory of Oncology in South China, Collaborative Innovation Center of Cancer Medicine, Guangzhou, China

**Keywords:** brain metastasis, differentiated thyroid cancer, RAIT, skull metastases, neurosurgery

## Abstract

**Background:**

Brain metastasis from differentiated thyroid cancer has followed a similar increasing trend to that of thyroid cancer in recent years. However, the characteristics and treatments for brain metastases are unclear. The aim of this study was to understand this disease by analyzing patients with brain metastases from differentiated thyroid cancer (DTC).

**Methods:**

Between 2000 and 2020, the database of the Sun Yat-sen University Cancer Center was searched for differentiated thyroid cancer patients. We identified a cohort of 22 patients with brain metastases. The characteristics of the patients, histological features, treatments, and time of death were reviewed. The overall survival (OS) rate was calculated using the Kaplan Meier method. Survival curves of different subgroups were compared according to baseline characteristics and treatments received.

**Results:**

A total of 22 (1.09%) out of 2013 DTC patients in the Sun Yat-sen University Cancer Center database were identified as having brain metastases. The overall median survival time was 17.5 months (range from 1–60 months) after diagnosis of brain metastasis. Performance statue (PS), tumor site, and neurosurgery impacted survival, according to Kaplan-Meier analysis. Prognosis of skull metastasis was superior to that of intracranial types. Neurosurgery was the only type of treatment that had an impact on patient OS.

**Conclusions:**

Brain metastasis from differentiated thyroid cancer has a poor prognosis. However, it can be improved by comprehensive treatment. PS of the patients can greatly affect survival. Skull metastases have improved prognosis over intracranial types. Radioiodine therapy (RAIT) appears to effectively improve the prognosis of patients with skull metastases from DTC.

## Introduction

Brain metastases (BMs) originating from differentiated thyroid cancer (DTC) are considered rare, occurring in approximately 1% of DTC (1, 2). However, the actual number may be higher; this is because nowadays, more asymptomatic lesions are found by systematic cerebral imaging before prescription of tyrosine kinase inhibitors (TKIs). On the other hand, owing to the rapid increase in the incidence of thyroid cancer, the rate of distant metastases in thyroid cancer is also rapidly increasing. In the literature, there are only a few relatively large retrospective studies with more than 10 cases (3–8). Management of patients with BM of DTC origin is unclear, with questions remaining regarding indications for neurosurgery, external beam radiotherapy (EBRT), radioiodine therapy (RAIT), stereotactic radiosurgery (SRS), TKIs, or whole-brain RT (WBRT).

The importance of neurosurgery and SRS for brain metastases has been consistently recognized, patients who had neurosurgery or SRS had remarkably better survival than those who did not (**Table 1**). However, the role of RAIT remains unclear. There is evidence to show that disease-free and total survival is improved by RAIT in patients with lymph node and/or distant metastases (9, 10). But the prognostic benefit of RAIT is still inconclusive for patients with metastatic differentiated thyroid cancer; for those with brain metastases, RAIT seems to have an effect.

**Table 1:** Summary of Case Series (>10 Patients) of BRAIN Metastases from DTC.

Study	Numbers of patients with BM from DTC	OS after BM diagnosis(months)	OS after BM and neurosurgery or SRS (months)
current study	22	17.5	34
Chiu et al. (3)	24	4.7	16.7
Samuel et al. (4)	15	6	
McWilliams et al. (5)	14	17.4	20.8
Bernad et al. (6)	12	20.8	37.4
Henriques et al. (7)	21	7.1	11.9
Jinhyun Choi et al. (8)	37	8.8	30.7

Skull metastasis is a special type of brain metastasis that invades the bony skull, but also the meninges and some functional areas of the brain. Compared with intracranial metastasis, it is rare. Skeletal deposits of neoplasm pose special hazards for fracture and, when adjacent to the central nervous system, neurologic impairment. A case report found that those patients with skull metastases appear to have a better prognosis compared to those with intracranial metastases (11). However, case reports can only offer limited information. In the current cohort, we improved our understanding of this special entity.

The management of brain metastatic DTC is very difficult, and it is necessary to glean more information regarding it. To our knowledge, the current study contains a relatively larger number of cases than can be found elsewhere in the published literature. The aim of this study was to review our experience with BM of DTC origin over the last 20 years in a large cancer center, in order to improve our understanding of this rare entity.

## Methods and Materials

Within the Sun-Yat Sen University cancer central database, we searched for patients with DTC and BMs during the period 2000-2020. The inclusion criteria met the following two points: 1. a confirmed history and pathology of DTC; 2. computed tomography (CT) or magnetic resonance imaging (MRI) showing brain lesions, with pathology having been confirmed as thyroid follicular carcinoma or papillary carcinoma; If points 1 + 2 were met, and primary carcinoma of other organs was excluded, the case was recruited.

Data of the recruited patients, including their follow-up data was collected. Among 2013 patients with DTC (surgeries between 1 January 2000 and 31 December 2020 and follow-up until 31 December 2020), 22 patients with BM were found and included in the current cohort. Medical records were reviewed and the following data was prepared: patient sex and age (at the DTC diagnosis and at the BM diagnosis), pathology, mode of presentation and radiologic features of BM, patient performance status (PS) [according the World Health Organization (WHO) classification at BM diagnosis], treatment modalities (neurosurgery, SRS, WBRT, RAIT, radiotherapy, chemotherapy), interval years, and patient outcome. In our series, there were five patients with skull metastases, which contained the largest cases of skull metastases. Therefore, patients with brain metastases were divided into an intracranial group and a skull group, according to the general location of the tumor to explore the differences in their characteristics and prognosis.Neurosurgical procedures performed in our center met the following conditions: for a single metastatic tumor, we performed surgery or SRS; for multiple metastatic tumors, but PS<2, neurosurgery was also considered. Pathology was confirmed by an expert thyroid pathologist who was blind to our study. Follow-up occurred until 31st December 2020. Internal review board approval was obtained.

Statistical Package for Social Sciences (SPSS) version 26.0 (IBM SPSS, Armonk, NY, USA) was used for statistical analysis. Overall survival (OS) after BM was calculated using the Kaplan-Meier method, and survival time was measured from the diagnosis of BM to the date of death or last follow-up. All causes of death were considered. Survival curves for various subgroups of patients according to baseline characteristics (age ≥60 or age<60, PS ≥2 or PS<2, lesion numbers of BM, tumor site or presence of neurologic symptoms at BM diagnosis), treatments received (neurosurgery, WBRT, SRS, chemotherapy, RAIT) and pathological features (tumor size, lymph nodes metastases, vascular Invasion, BRAF mutation) were compared using a two-sided log-rank test (p-value <0.05 was considered significant). Those factors with a p-value <0.1 were then gathered and included in the multivariate COX analysis in order to determine which had an independent impact on survival (p-value <0.05 was taken to be significant). At the same time, we performed a survival analysis on various treatments that may affect progression-free survival (PFS).

## Results

### Patient Characteristics and Histology

The basic conditions of the patients are shown in **Table 2**. Of the 22 patients, 12 were male and 10 female (male/female ratio = 1.2:1). The mean patient age was 54.5 years (range: 39–76 years) at BM diagnosis. Fourteen patients presented other previous and/or synchronous distant metastases: lung (12), bone (6), liver (1), skin (1). The mean interval time between diagnosis of the first metastasis and BM was 2 years (range: 0–20 years) for these patients.

**Table 2:** Individual clinical findings of patients with BRAIN metastases from DTC.

Patient (years At BM diagnosis)	Sex	Pathology	Age at BM diagnosis(years)	PS (ECOG) at BM diagnosis	Interval years	Coexistent distant Metastasis	Location of BM/Numbers of the lesion (size)	Neurosurgery or not	Overall survival (months)	Other Treatment
Patient1 (2002)	Male	unknown	65	1	synchronous	lung, bone	Intracranial/M(2cm×3cm)	NO	13(death)	WBRT+CT
Patient2 (2004)	Male	P	54	0	2	lung, bone	IntracranialM(3cm×2cm)	NO	19(death)	SRS+WBRT+RAIT+CT
Patient3 (2005)	Male	P	52	2	1	lung, bone	Skull/S(3.8cm×5.5cm)	YES	34(death)	SRS+RAIT+CT
Patient4 (2007)	Female	F	68	4	synchronous	NO	Intracranial/M(3cm×2cm)	NO	1(death)	Palliative CT
Patient5 (2008)	Male	P	56	0	4	lung, bone, liver	Skull/M(2.6cm×2.1cm)	YES	52(death)	WBRT+CT
Patient6 (2009)	Male	F	45	1	synchronous	lung	Skull/S(5cm×5cm)	YES	60(death)	RT+RAIT
Patient7 (2010)	Male	P	70	3	0.5	lung, skin (foot)	Intracranial/M(2cm×1.5cm)	NO	3(death)	SRS+Palliative CT
Patient8 (2014)	Female	P	42	1	synchronous	lung	Intracranial/M(1.7cm×1.5cm)	YES	24(death)	Sorafeni
Patient9 (2014)	Male	F	45	3	0.5	lung	Intracranial/M(0.6cm×0.8cm)	NO	10(death)	CT
Patient10 (2014)	Male	F	60	3	10	NO	Intracranial/S(2cm×2cm)	YES	1(death)	Palliative CT
Patient11 (2015)	Female	unknown	53	1	synchronous	NO	Skull/S(2cm×2cm)	NO	46(Alive)	RAIT
Patient12 (2015)	Female	P	41	1	synchronous	NO	Intracranial/S(2cm×2.6cm)	YES	25(death)	RT+RAIT
Patient13 (2016)	Female	P	75	3	20	lung	Intracranial/M(2cm×2.1cm)	NO	5(death)	Palliative RT
Patient14 (2017)	Male	P	27	1	3	lung	Skull/S(4cm×4cm)	NO	30(Alive)	PD-1+RAIT
Patient15 (2017)	Female	P	48	2	synchronous	NO	Intracranial/M(2cm×2.1cm)	YES	36(Alive)	SRS+RT+RAIT
Patient16 (2017)	Male	P	56	2	synchronous	lung	Intracranial/S(2cm×2 cm)	YES	12(death)	RT+CT
Patient17 (2018)	Male	F	42	1	synchronous	lung	Intracranial/S(2cm×2 cm)	YES	28(Alive)	RAIT
Patient18 (2018)	Female	F	66	2	synchronous	NO	Intracranial/S(4cm×3 cm)	YES	24(Alive)	RT
Patient19 (2019)	Female	P	39	1	synchronous	NO	Intracranial/S(1cm×1 cm)	YES	16(Alive)	RT
Patient20 (2019)	Female	P	55	1	12	bone	Intracranial/S(3cm×3 cm)	YES	16(Alive)	RT+RAIT
Patient21 (2019)	Male	P	76	2	5	NO	Intracranial/M(3.4cm×3.5 cm)	NO	10(Alive)	Palliative RT
Patient22 (2020)	Female	P	55	1	6	lung, bone	Intracranial/S(2.5 cm×3.5 cm)	YES	6(Alive)	Sorafeni

P, papillary; F, follicular; PS, performance status; BM, brain metastasis; CT, chemotherapy; WBRT, whole brain radiotherapy treatment; RT, radiotherapy treatment; RAIT, Radioiodine Therapy; M, multitude; S, single.

Fourteen patients had papillary carcinoma, six patients had follicular carcinoma and two patients were unknown. Stages were pT0-2 for 12 patients, pT3-4 stage for eight patients, and unknown for two patients. Nodal involvement was present for 17 patients, absent for three patients, and unknown for two patients. Twelve patients had histological confirmation that the brain lesions were of thyroid origin through biopsy or resection. Nine cases showed vascular invasion, while 10 cases showed no invasion. Three cases were unknown. Necrosis was absent in all cases. Moderate nuclear atypia was observed in 6 cases, where as in 14 cases it was severe. BRAF mutation was found in 9 cases, while 7 cases were not. Six cases were unknown. Seven of 9 (77.8%) cases with BRAF mutation showed vascular invasion, which occurred in only 2 of 7 (28.6%) cases of patients without BRAF mutation.

### Clinical Features of BM

The mean number of BM was 2.3 (range: 1-6). Eleven patients had a single BM while the remainders had multiple BMs. For 15 patients, BMs were revealed by neurologic symptoms, including headaches, nausea, motor or sensory deficits, ataxia, aphasia, confusion, and epileptic seizures. For the remaining seven patients, BMs were diagnosed by chance and presented no symptoms. Diagnoses were made by systematic cerebral imaging before TKI prescription for three patients, by iodine scans for four patients, by PET/CT scans for two patients, by magnetic resonance imaging (MRI) for three patients, and by enhanced computed tomography (CT) for three patients. WHO PS at BM diagnosis was good (<2) for 14 patients and poor (≥2) for eight. For 16 patients, CT images or MRI were available and reviewed. Five patients had metastatic tumors where the main tumor was located in the skull; the remaining 17 cases were located intracranially.

### Treatment

All patients received comprehensive treatments. Twelve patients underwent thyroid surgery. Ten patients underwent neurosurgery for complete removal of the tumor; four patients underwent SRS instead of neurosurgery. Ten patients underwent radiotherapy including IMRT and 3D-RT; three patients underwent WBRT; and nine patients received chemotherapy.

### Survival

**Figure 1:**
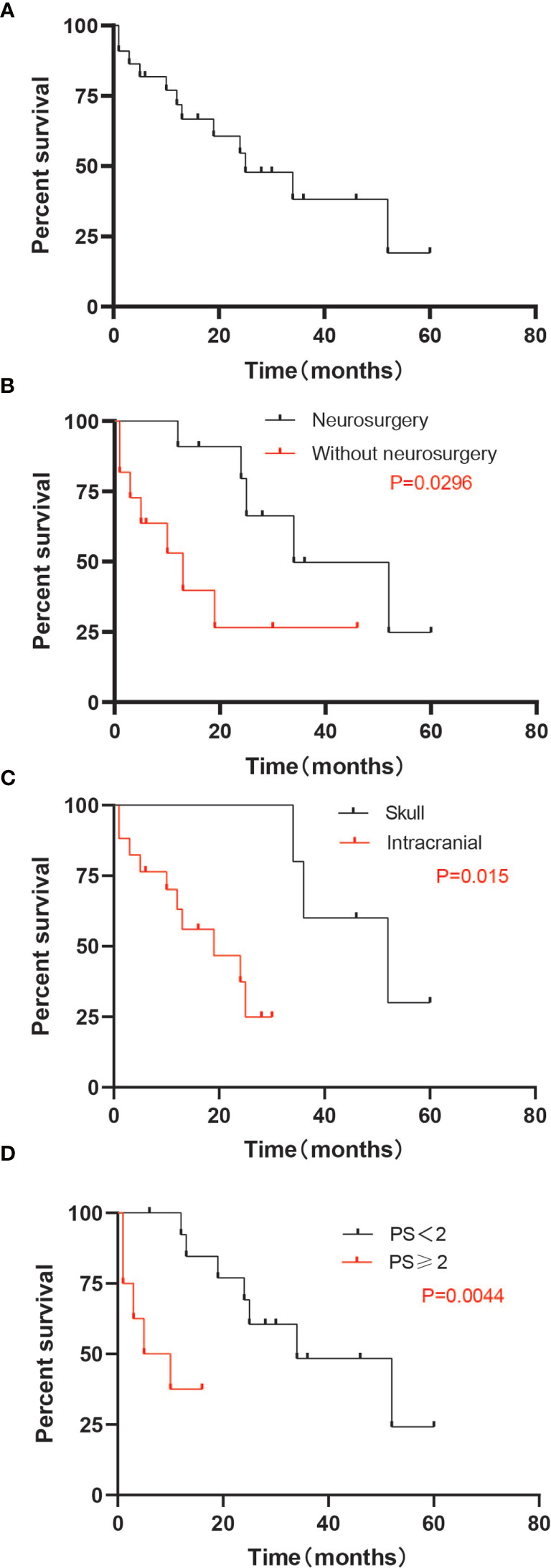
Median OS. **(A)** Median OS for all patients (25 months). **(B)** Median OS by group of patients with or without neurosurgery. The OS was 40.6 months with neurosurgery, and 18.4 months without neurosurgery (p = 0.0296). **(C)** Median OS by group of patients whose lesions were in the skull or intracranial. The OS was 51.6 months in patients whose lesions were located in skull, and 17.6 months for intracranial lesions (p = 0.015). **(D)** Median OS by group of patients with good PS (<2) or poor PS (≥2). The OS was 40.6 months with PS<2 and 18.4 months for PS ≥2 (p = 0.0044). OS, overall survival; PS, performance status.

At the time of the last follow-up, 11 patients had died and 11 remained alive, with an overall survival time ranging from 1 to 60 months (**Table 2**). Among the 11 patients who died, the causes of death were attributed to tumor progression, including brain metastases and other metastatic sites. Seven patients died of brain tumor progression, and two patients died of lung tumor progression. For two patients, the cause of death was unknown, as data was missing in their files. Median OS after BM was 17.5 months (range 1-60 months). Survival for 15 patients was over 1 year. The longest OS reached 60 months; OS at 1 and 2 years were, respectively, 68.2% and 45.5%. PS, tumor site of patients, and neurosurgery had an impact on overall survival rate as assessed using the Kaplan-Meier method (p-value < 0.05). The survival curve is shown in **Figure 1**. No impact of WBRT, RIT, chemotherapy, sex, interval years, pathology, thyroid surgery, pathological features(tumor size, lymph nodes metastases, vascular Invasion, BRAF mutation), any other distant transfer lesions, or presence of neurologic symptoms at BM diagnosis was found. Multivariate COX analysis was performed and it was found that PS was an independent prognostic factor of BM from DTC (p<0.007) (**Table 3**). Median OS was 38.7 months if PS was <2 and only 18.3 months if PS was ≥2. Median OS was 34 months if neurosurgery were performed and 13 months in the absence of neurosurgery (p=0.0296). To our surprise, tumor site had a substantial impact on survival. The results showed that the first 5 patients with the longest survival time all belonged to the skull metastases group. Median OS was 51.6 months if the tumor site was located in the skull and only 17.6 months if the tumor site was located intracranially (p=0.015). We found that 12 cases are able to meet our enrollment requirements(the other 10 patients were unable to count the PFS due to the lack of image data and any other reasons). We evaluated different type of therapeutic approaches(WBRT, RIT, chemotherapy, SRS, RT, neurosurgery) and found that SRS and neurosurgery have an impact on PFS (p<0.05) (**Table S1**).

**Table 3:** Kaplan-Meier (Log-Rank Test) and Multiple Cox Regression Analysis of Prognostic Factors for 22 Patients with Brain Metastases of DTC.

Factors	Numbers of patients	OS (months)(median ± 95%CI)	P (K-M)	P(COX)
Sex				
Male	13	24.2 ± 6.2	0.096	0.651
Female	9	42.4 ± 7.8		
Pathological				
Papillary	13	31.4 ± 5.7	0.874	
Follicular	7	21.6 ± 10.6		
Thyroid operation				
No	6	28.8 ± 7.0	0.976	
Yes	16	30.1 ± 6.1		
Neurosurgery				
No	11	18.41 ± 5.8	0.030	0.501
Yes	11	40.6 ± 5.9		
Other distance				
No	8	29.5 ± 7.2	0.558	
Yes	14	28.8 ± 5.9		
Symptom				
No	7	32.3 ± 5.7	0.499	
Yes	15	29.0 ± 6.4		
Age(years)				
≤55	14	37.2 ± 5.6	0.055	0.862
>55	8	13.4 ± 4.5		
Radiotherapy Treatment				
No	10	26.0 ± 6.3	0.606	
Yes	12	33.0 ± 6.4		
Radioiodine Therapy				
No	11	23.64 ± 5.6	0.272	
Yes	11	36.6 ± 6.8		
Lesions site				
Intracranial	17	17.6 ± 2.7	0.015	0.935
Skull	5	51.6 ± 4.5		
Lesions number				
Single	13	37.1 ± 6.0	0.084	0.111
Multiple	9	18.3 ± 6.3		
PS				
<2	8	38.7 ± 5.4	0.004	0.007
≥2	14	8.5 ± 2.3		

## Discussion

The prevalence of BM among patients with metastatic thyroid cancer is estimated to be 4.5–18% (1, 2). With an increase in the number of physical examination centers, endocrinologists, and surgeons, and the progress taking place in imaging, the diagnosis of thyroid cancer is increasing, and the diagnosis of brain metastatic tumors is also increasing year by year (13). Management of these patients remains difficult because treatment for BM has not improved over recent decades, and has been mostly based on experience, with no uniform guidelines. Under such conditions, it is hard to understand this rare entity.

In our series, 12 of 22 patients were male and 10 female (male/female ratio = 1.2:1); given that the sex ratio for thyroid cancer is 3:1 to women, men seem to be more prone to brain metastases. The mean age of patients was 54.5 years (range: 39–76 years) at BM diagnosis. Fourteen patients presented other previous and/or synchronous distant metastases: lung (12), bone (6), liver (1), skin (1).

In the current cohort, with a median OS after BM of 17.5 months, outcomes of brain metastatic patients from DTC appeared poor. However, this number is higher than in most reported literature (**Table 1**). This may be owing to the fact that patients in our center had received relatively comprehensive treatments. On the other hand, patients with medullary or anaplastic carcinomas, which had a poorer prognosis compared to DTC, were excluded, while most other reports did not do this. Interval time did not have a significant impact on OS. And we also discovered that there was no difference in OS between patients with BMs detected during follow-up and synchronous BMs. However, we found that the recurrence rate of patients with synchronous BMs was 8/11 (72.7%) *versus* 3/11 (27.2%) with BMs detected during follow-up. “Synchronous BMs” seems to be associated with poor prognosis.

There are retrospective studies regarding BM from DTC (number of cases ≥10) with inherent bias (3–8). Chiu et al. (3) and McWilliams et al. (5) emphasized the importance of neurosurgery for brain metastasis of thyroid carcinoma, they reported a survival of 16.7 and 20.8 months after surgery versus 3.4 and 2.7 months without neurosurgery. In the current series, median OS was 34 months if neurosurgery was performed and 13 months in the absence of neurosurgery (p = 0.01). We further support the significance of neurosurgery. Although poor general condition usually indicates a contraindication for craniotomy, two patients were in poor general condition (PS ≥2) when they received neurosurgery. One patient lived for 36 months while the other lived for 28 months and is currently still alive; they benefited from the neurosurgery. So it is pertinent to suggest that the conditions of neurosurgery for tumor removal should be expanded.

Patients with a single BM seem to have a better outcome than patients with multiple metastases [OS of 12 months vs. 3.7 months in the study by Chiu et al. (3)]. However, we saw no difference in the patients with single metastases compared with those with multiple metastases. SRS treatment is now available for BM from lung, breast, colorectal, and renal cancers, or melanomas (12, 14). Kim et al. (15) (nine cases of brain metastatic) and Bernad et al. (6) (15 cases of brain metastatic) found that SRS treatment can have a huge impact in patients with brain metastases; OS in their studies reached the highest published, with 33 and 37.4 months, respectively. In our series, only four patients received SRS treatment and their OS was similar to those patients who did not received SRS treatment. However, we found that SRS had an impact on PFS (p<0.05). It could improve the local control rate of the brain tumor. In those four patients, no recurrence of brain metastasis was found after SRS treatment, they all died from the progression of other distant metastases.

In the current series, there were 14 cases of papillary carcinoma and six cases of follicular carcinoma. There was no difference between them in OS. However, McWilliams et al. (5). reported that follicular carcinoma (OS = 8.3 months) was thought to have a poorer prognosis than papillary carcinoma (OS = 23.6 months). We suggest that this result is very partial, because the study only included two cases of thyroid follicular carcinoma. Recent studies have established a strong association of BRAF mutation with aggressive clinicopathologic characteristics of the primary PTC, including extrathyroidal extension, lymph node metastasis, histologic subtypes with a poorer prognosis (e.g., tall cell variant PTC),and advanced disease stages, and disease persistence/recurrence (16). In the current series, seven of 9 (77.8%) cases with BRAF mutation showed vascular invasion, which occurred in only 2 of 7 (28.6%) cases of patients without BRAF mutation. BRAF mutation seems to be a factor promoting DTC metastasis. Previously, WBRT was reported to improve the local control rate through eliminating microlesions of BM after neurosurgery (17–19). In the current series, three patients received WBRT after neurosurgery; two of them showed a good local control rate, but both suffered from neurotoxicity. Therefore, the risk of long-term neurotoxicity of WBRT should be taken into consideration (20, 21).

Skull metastatic tumors are unique; although they occur at the bone-brain junction, they can invade the meninges and some functional areas of the brain. Therefore, we suggest that skull metastatic tumors are rare and should be treated differently. Hence, we divided brain metastases into skull metastases and intracranial metastases according to the general location of the tumor and tried to identify differences between them. Interestingly, the current series showed that the first five patients with the longest survival time all had skull metastases. Median OS was 46 months if the lesions were located in the skull and only 13 months if the lesions were located intracranially. Misaki (22) found that the blood brain barrier (BBB) blocked the accumulation of chemotherapeutic drugs in tumors and weakened the irradiation. RAIT after neurosurgery seemed safer because the absence of the skull could avoid brain tissue edema caused by RAIT or thyroid stimulating hormone (TSH) suppressive therapy, which stimulate metastatic growth and abrupt clinical deterioration (11). On the other hand, the BBB and the meninges can protect normal brain tissue from high concentrations of chemotherapeutic drugs and high-dose radiation. In addition, because of the existence of the BBB, the concentration of TKIs such as sorafenib and immunotherapy drugs such as PD-1 in skull tumors will be higher than those in intracranial tumors. Thus, we suggest that both the prognosis and quality of life associated with skull metastases are better than for intracranial ones.

Bal (23) found that RAIT is a good factor for long-term outcome of patients with BM from DTC. Although we found RAIT had no impact on the survival of the current cohort of patients with brain metastasis, according to the Kaplan-Meier method. Interestingly, we noted that all the patients with the skull metastases received RAIT, that they were less likely to have fatal side effects of RAIT such as brain edema, and had longer OS. There were five patients with intracranial metastases in our series who received RAIT; their median OS was only 11.5 months, which was much lower than for patients with skull metastases whose median OS was 51.6 months. Patients with skull metastases appeared to show a greater benefit following RAIT.

**Figure 2:**
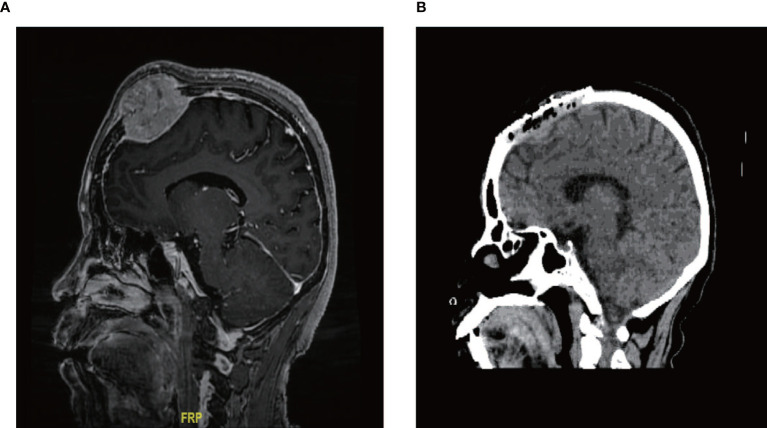
Typical case of skull metastasis. **(A)** Brain metastases were located in the skull, with a clear boundary with the meninges at diagnosis. **(B)** no recurrence was found after neurosurgery and RAIT.

In our series, one male patient with the longest survival time (60 months) had a lesion that was located in the parietal bone and was 5 cm× 5 cm in size (**Figure 2A**). After neurosurgery, he received high-dose (16 Gbq) RAIT and TSH suppressive therapy, and experienced no fatal side effects such as cranial hypertension. His skull tumor disappeared and showed no recurrence during follow-up (**Figure 2B**).

Sorafenib was approved by the US Food and Drug Administration and the European Medicines Agency to improve progress-free survival in patients with ADTC or PDTC (24–26). Sheu (27) reported that two patients with DTC craniocranial metastases were treated with Lenvatinib combined with RAIT, which successfully improved their quality of life. Cristiane J (28) found that the median survival time after diagnosis of brain metastases was 19 months and the use of TKIs improved the survival rates. In the current series, one patient who treated with sorafenib after neurosurgery is now still alive. The quality of her life was significantly improved. However, there was no evidence that this treatment could improve the overall survival of patients, because only two patients were treated with sorafenib in the current cohort, it was hard to evaluate its effectiveness. Further study is needed.

**Figure 3:**
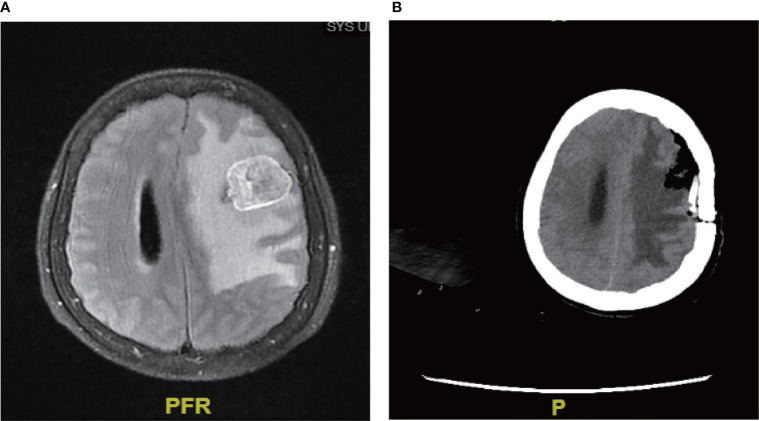
Neurosurgery combined with PD-1. **(A)** Brain metastases were located intracranially. **(B)** Following neurosurgery combined with PD-1, no recurrence was found after 6 months.

In our cohort, a 27-year-old female patient received neurosurgery combined with PD-1 and RAIT. She had no recurrence after 6 months (**Figure 3**), her intracranial tumor was controlled at the last follow-up, and the patient now has a high quality of life. Neurosurgery combined with PD-1 and RAIT seemed to be very helpful for the treatment of this rare entity.

Our results need to be interpreted with caution owing to their retrospective character, the low number of patients, and the long period of study with inherent changes of practice. However, this study remains interesting because, with 22 patients, it is one of the biggest found in the literature. Moreover, we draw a series of meaningful conclusions by the survival analysis of 22 patients, which could help to improve our understanding of this rare entity.

## Conclusions

BM from DTC has a poor prognosis, however, the prognosis can be improved if it is treated aggressively. In guidelines published in 2009, the American Thyroid Association acknowledges the necessity and effectiveness of neurosurgery in the final stages of thyroid cancers such as central nervous system metastases (29); our research confirms those findings. The most important prognostic factor for selecting patients for these aggressive treatments appears to be a good PS (<2). Although BMs from DTC are rare, for those patients with headache or other central nervous symptoms, the existence of BMs should be considered, as the proportion of brain metastases in distant metastasis is not low. We cannot deny the effect of RAIT, it had a significant impact on patients whose lesions were located in the skull. TKIs and immunotherapy such as PD-1 showed good prospects. Reviewing the outcomes of these patients should enable us to better improve patient prognosis in the era of modern treatments.

## Data Availability Statement

The original contributions presented in the study are included in the article/**Supplementary Material**. Further inquiries can be directed to the corresponding author.

## Ethics Statement

Written informed consent was obtained from the individual(s) for the publication of any potentially identifiable images or data included in this article.

## Author Contributions

AY designed the main idea of the experiment. TW performed the main work of the research, and was a major contributor in writing the manuscript. All authors contributed to the article and approved the submitted version.

## Funding

All of the work was supported by the Science and Technology Planning Project of Guangdong Province, China (2014A020212100).

## Conflict of Interest

The authors declare that the research was conducted in the absence of any commercial or financial relationships that could be construed as a potential conflict of interest.

## Publisher’s Note

All claims expressed in this article are solely those of the authors and do not necessarily represent those of their affiliated organizations, or those of the publisher, the editors and the reviewers. Any product that may be evaluated in this article, or claim that may be made by its manufacturer, is not guaranteed or endorsed by the publisher.

## Abbreviations

BBB, blood-brain barrier; BM, brain metastasis; CT, computed tomography; DTC, differentiated thyroid cancer; EBRT, external beam radiotherapy; IMRT, intensity modulated radiation therapy; MRI, magnetic resonance imaging; OS, overall survival; PS, performance status; PD-1, programmed cell death-1; PFS, progression-free survival; RAIT, radioiodine therapy; SRS, stereotactic radiosurgery; TKI, tyrosine kinase inhibitor; TSH, thyroid stimulating hormone; WBRT, whole-brain radiotherapy.
