# Supplementary material for: Brain Metastases From Differentiated Thyroid Carcinoma: A Retrospective Study of 22 Patients
Source: Front Endocrinol (Lausanne). 2021 Sep 16;12:730025. doi: 10.3389/fendo.2021.730025 (PMC8481895; doi:10.3389/fendo.2021.730025)
Supplement: Supplementary file 1 [file Table_1.docx]

***TbaleS1. Kaplan-Meier (Log-Rank Test) for 12 Patients with Brain Metastases of DTC***

| Treatments | Numbers of patients | PFS (months)  （median±95%CI） | P（K-M） |
| --- | --- | --- | --- |
| Neurosurgery |  |  |  |
| NO | 9 | 7.4±2.1 | 0.042 |
| YES | 3 | 26.0±7.3 |  |
| CT |  |  |  |
| NO | 8 | 12.0±4.3 | 0.888 |
| YES | 4 | 13.5±7.2 |  |
| TKIs |  |  |  |
| NO | 10 | 9.9±3.5 | 0.130 |
| YES | 2 | 24.5±8.1 |  |
| SRS |  |  |  |
| NO | 8 | 6.3±1.3 | 0.043 |
| YES | 4 | 23.0±6.6 |  |
| RT |  |  |  |
| NO | 6 | 14.3±4.5 | 0.306 |
| YES | 6 | 10.6±6.5 |  |
| RAIT |  |  |  |
| NO | 8 | 11.7±4.4 | 0.964 |
| YES | 4 | 14.0±7.0 |  |
| WBRT |  |  |  |
| NO | 10 | 11.9±4.3 | 0.631 |
| YES | 2 | 16.0±3.0 |  |
| **CT=chemotherapy, TKIs= tyrosine kinase inhibitors, WBRT=whole brain radiotherapy treatment, RT=radiotherapy treatment,**  **RAIT= Radioiodine Therapy, SRS= stereotactic radiosurgery.** | | | |
